# Vaccination of health care workers in Romania

**DOI:** 10.1186/1471-2334-14-S7-P75

**Published:** 2014-10-15

**Authors:** Daniela Pițigoi, Liliana Lucia Preoțescu, Anca Streinu-Cercel, Maria Nițescu, Victoria Aramă, Alexandru Rafila

**Affiliations:** 1 Carol Davila University of Medicine and Pharmacy, Bucharest, Romania; 2 National Institute for Infectious Diseases "Prof. Dr. Matei Balş", Bucharest, Romania

## Background

We aimed to review existing regulations about HCWs vaccination in Romania and to identify the attitude and barriers to immunizations of HCWs through qualitative research.

## Methods

We collected information about HCWs vaccination policies and regulation implemented through laws and also data about specific vaccination (seasonal influenza, hepatitis B, measles, mumps, rubella and varicella). We conducted 5 focus groups with 39 participants (nurses, physicians, infection control personnel, public health and policy makers), as part of the HProImmune (Promotion of Immunizations for Health professionals in Europe) project activities, in order to understand the risk perception behaviors towards vaccination and barriers stopping HCWs from immunization. We followed for each focus group a specific discussion guide elaborated by the HProImmune project.

## Results

Each health facilities organize yearly influenza vaccination campaigns and HCWs received influenza vaccine at their work place, being covered from the Ministry of Health budget. No specific national recommendation regarding HCWs vaccination against varicella, pertussis, tetanus, diphtheria, poliomyelitis, meningoccocus exist in Romania. The main barriers included: insufficient information on benefits of vaccines, insufficient information on diseases and the risk of diseases, concerns about vaccine effectiveness and adverse events, lack of time, lack of clear national guideline concerning the HCW immunization. Dominant immunization enablers included: influence of educational programs (in school and at the workplace) and communication campaigns, importance of hospital epidemiologist, infection control personnel and occupational physicians. The majority of HCWs highlighted that the immunizations should be mandatory in health care facilities.

## Conclusion

Clear immunization policy and guidelines regarding occupational vaccination of HCWs in Romania, communication strategy and training programs are needed in order to increase confidence in vaccine and the vaccination coverage among HCWs.

